# Olaris Global Panel (OGP): A Highly Accurate and Reproducible Triple Quadrupole Mass Spectrometry-Based Metabolomics Method for Clinical Biomarker Discovery

**DOI:** 10.3390/metabo14050280

**Published:** 2024-05-11

**Authors:** Masoumeh Dorrani, Jifang Zhao, Nihel Bekhti, Alessia Trimigno, Sangil Min, Jongwon Ha, Ahram Han, Elizabeth O’Day, Jurre J. Kamphorst

**Affiliations:** 1 Olaris, Inc., 175 Crossing Boulevard Suite 410, Framingham, MA 01702, USA; mdorrani@myolaris.com (M.D.); jzhao@myolaris.com (J.Z.); nbekhti@myolaris.com (N.B.); atrimigno@myolaris.com (A.T.); eoday@myolaris.com (E.O.); 2 Seoul National University Hospital, 101, Daehak-ro, Jongno-gu, Seoul 03080, Republic of Korea; surgeonmsi@gmail.com (S.M.); jwhamd@snu.ac.kr (J.H.); ahramh@gmail.com (A.H.)

**Keywords:** biomarkers, clinical metabolomics, HILIC, hydrophilic interaction liquid chromatography, triple quadrupole mass spectrometry, U-^13^C-metabolite internal standard

## Abstract

Mass spectrometry (MS)-based clinical metabolomics is very promising for the discovery of new biomarkers and diagnostics. However, poor data accuracy and reproducibility limit its true potential, especially when performing data analysis across multiple sample sets. While high-resolution mass spectrometry has gained considerable popularity for discovery metabolomics, triple quadrupole (QqQ) instruments offer several benefits for the measurement of known metabolites in clinical samples. These benefits include high sensitivity and a wide dynamic range. Here, we present the Olaris Global Panel (OGP), a HILIC LC-QqQ MS method for the comprehensive analysis of ~250 metabolites from all major metabolic pathways in clinical samples. For the development of this method, multiple HILIC columns and mobile phase conditions were compared, the robustness of the leading LC method assessed, and MS acquisition settings optimized for optimal data quality. Next, the effect of U-^13^C metabolite yeast extract spike-ins was assessed based on data accuracy and precision. The use of these U-^13^C-metabolites as internal standards improved the goodness of fit to a linear calibration curve from r^2^ < 0.75 for raw data to >0.90 for most metabolites across the entire clinical concentration range of urine samples. Median within-batch CVs for all metabolite ratios to internal standards were consistently lower than 7% and less than 10% across batches that were acquired over a six-month period. Finally, the robustness of the OGP method, and its ability to identify biomarkers, was confirmed using a large sample set.

## 1. Introduction

Metabolomics can be defined as the large-scale study of small molecules (metabolites) present in biological systems and is a relatively recent addition to the field of ‘omics’ research, which includes genomics, transcriptomics, and proteomics. Metabolites are dynamically regulated in response to a variety of processes that include endogenous metabolism and signaling, metabolism of the microbiome, diet, and exposure to the environment and drugs [1]. Consequently, metabolomics sits at the nexus of genetic and environmental impact and offers a unique real-time readout of the physiological or pathological state in that moment of time. However, adoption of metabolomics has been relatively slow compared to some of the other omics. This is in part due to the limited reproducibility of metabolite coverage and abundance [2]. Additionally, processing of metabolomics data, especially high-resolution data, still requires specialist knowledge, limiting its integration into clinical labs.

Nevertheless, there is tremendous potential for metabolites to provide clinical value for a variety of diseases [3]. Indeed, many of the top diagnostics are already based on metabolite readouts. This includes glucose for diabetes, cholesterol and other lipids for cardiovascular disease, and creatinine for kidney function [4,5]. Examples of how metabolomics can provide further clinical value include the following: diagnosis of elusive inborn errors of metabolism (IEM) [6], improved detection of insulin resistance and type 2 diabetes [7,8], the ability to predict cardiovascular risk in patients with coronary artery disease (CAD) [9], improved diagnosis of neurological disorders such as Parkinson’s disease [10,11,12], and the improved ability to diagnose certain types of cancer and monitor treatment response [13,14,15]. Thus, metabolomics, on its own or used in conjunction with other omics, is uniquely positioned to make significant contributions to elucidating molecular mechanisms of disease as well as identifying new biomarkers to accelerate drug programs, improve diagnostics, and facilitate patient sub-setting (i.e., precision medicine).

Metabolomics has been rapidly growing mainly due to advancements in analytical techniques, such as NMR and mass spectrometry (MS), the two main platforms for profiling metabolites in samples [16,17]. Among the MS-based techniques, untargeted metabolomics using high-resolution accurate mass spectrometry (HRAMS) has been widely adapted as an analytical tool for characterizing the metabolome due to its potential to measure the abundance of thousands of ions in a single run, including unknowns [18,19,20,21]. HRAMS instruments have been especially powerful in studies involving the use of stable isotope labeled nutrients to ‘trace’ metabolic activity and in exploratory studies where the focus is on identifying metabolic differences between conditions of interest, including unknown signals. It is important to note, however, that structural elucidation of unknowns is a highly specialized endeavor that is complicated by the presence of myriad isotopes, adducts, in-source fragment ions, and artifacts [22,23]. The omnipresence of these signals also typically leads to a gross overestimation of the actual number of metabolites that are measured in a LC-MS run. In fact, the number of metabolomic features in a sample has been reported to exceed the count of unique metabolites by one to two orders of magnitude [22,24].

For the analysis of clinical samples, targeted metabolomics using triple quadrupole (QqQ) MS, especially in combination with stable isotope-labeled standards, may provide a powerful solution for several reasons. First, with continued innovations in QqQ MS technology, scan speeds have increased to a point where the number of metabolites that can be profiled in a single LC-MS run is comparable to HRAMS. This makes comprehensive coverage of the known metabolome that can be measured by LC-ESI-MS tractable [25]. Second, QqQ MS provides superior sensitivity and linear dynamic range, which, in combination with stable isotope-labeled standards, provides the accuracy, precision, and consistency in metabolome coverage, all of which are required for successful clinical metabolomics application [18,21]. Finally, the cost-effectiveness and ruggedness of the QqQ instruments, along with the simplified data processing pipeline, make it feasible to process very large datasets and implement the method in clinical laboratories without expert metabolomics experience. Recent examples of QqQ-based metabolomics include a seminal work by López-Hernández et al., in which 180 urinary metabolites in a set of samples from both healthy and ill newborns admitted to the NICU were measured to investigate metabolic signatures related to life-threatening perinatal complication [26]. In another study, QqQ-based metabolomics was used to analyze a total of 184 metabolites in a large European population in order to identify potential biomarkers for cardiovascular risk assessment [27].

Here, we introduce the Olaris Global Panel (OGP), a QqQ MS-based metabolomics method covering approximately 250 metabolites (at the time of writing) from all major metabolic pathways. It addresses the limitations in accuracy and reproducibility hampering wide-scale adoption in a clinical setting, while at the same time maximizing ease of use. As outlined in this manuscript, central to the success of the method are (1) the careful selection and optimization of the chromatographic and MS settings and (2) the use of U-^13^C internal standards. The OGP achieves high precision for samples acquired across a six-month timespan, as well as high accuracy based on linear range assessments and a comparison to 2D-NMR, the gold standard in data accuracy. Finally, the method’s robustness and ability to identify clinically relevant metabolite signatures is demonstrated using a clinical cohort of urine samples.

## 2. Materials and Methods

### 2.1. Chemicals and Material

LC-MS grade acetonitrile (ACN) and methanol (MeOH) were purchased from VWR analytical (Radnor, PA, USA). LC-MS grade water was obtained from Thermo Fisher Scientific (Waltham, MA, USA). Metabolite standards were purchased from MetaSci, Inc. (Toronto, ON, Canada) or Sigma Aldrich (St. Louis, MO, USA). Stable isotope labeled (ISO1, U-^13^C, 98%) and unlabeled metabolite yeast extract (ISO1-UNL) were obtained from Cambridge Isotope Laboratories, Inc. (Andover, MA, USA). Pooled commercial urine used for making samples with different specific gravities (SG) was obtained from UTAK (UTAK, Valencia, CA, USA). All other pooled commercial urine samples used in this study were purchased from Innovative Research (Innovative Research Inc., Novi, MI, USA). Specific gravity (SG) of urine samples was measured using a refractometer (Palm Abbe Digital Refractometer, MISCO, Solon, OH, USA).

### 2.2. U-^13^C Metabolite Yeast Extraction

The dried U-^13^C metabolite yeast extract was solubilized in 2 mL of water: MeOH (1:1 *v*/*v*) by vigorously shaking the Falcon tube by hand with intermittent high-speed vortexing until the pellet was completely dissolved. The solution was then centrifuged at 20 °C for 5 min at 4000 rcf. Subsequently, the supernatant was carefully transferred to microcentrifuge tubes and kept at −80 °C until use.

### 2.3. Sample Preparation

Urinary metabolites were extracted as previously reported with some modifications [28]. In the first step, a Working Internal Standard (WIS) solution was prepared by adding 1 volume of U-^13^C metabolite yeast extract, prepared in Section 2.2, to 4 volumes of an ACN: MeOH (1:1 *v*/*v*) solution. Prior to analysis, the SGs were pre-adjusted to ~1.02 for all urine samples above this density. Next, 25 µL of urine sample was added to 75 µL WIS solution in an microcentrifuge tube. The sample was vortexed for 60 s and centrifuged for 10 min at 4 °C and 13,500 rpm. Subsequently, 80 µL of the supernatant was transferred to a LC-MS vial and stored at −20 °C until analysis.

For linear response assessment, a pooled commercial urine sample (SG = 1.0097) was diluted (2×, 5×) with water or concentrated 2, 4, and 6 times by lyophilizing 5 mL aliquots of urine overnight and resuspending them in varying volumes of water to obtain different SG values of 1.0187, 1.0275, and 1.0382.

### 2.4. Clinical Samples

To examine the performance of the OGP for the detection of clinically relevant metabolite signatures, a set of 225 urine samples from patients having undergone a kidney transplant either two weeks or a year prior were prepared and analyzed according to Section 2.3 and Section 2.5, respectively. A quality control sample (QC) was identically prepared after pooling 10 µL aliquots of each study sample. LC-MS acquisition of the patient samples was performed in a single batch and in a randomized order interspersed with a pooled urine QC sample run at the beginning of the batch, after every 10 study samples and at the end of sequence to monitor the analytical quality of the run. To confirm linear response of the metabolites, a QC sample dilution series (1×, 2×, 4×, and 8× diluted) were run at the beginning of the batch. Additionally, a pooled commercial urine sample (reference sample) was prepared and run in triplicate at the beginning of each sequence to monitor instrument performance and evaluate reproducibility both within and across batches. Finally, blanks were run at the beginning and the end of the sequence to rule out carryover.

### 2.5. Instrumentation and LC-MS Data Acquisition

Targeted mass spectrometric analysis of urinary metabolites was carried out using a Thermo Fisher Scientific Altis MD with a Thermo Fisher Scientific Vanquish Flex UHPLC system. All samples were analyzed in randomized order and evenly interspersed by QC samples by injecting 2 µL of the sample on a Waters Atlantis Premier BEH Z-HILIC VanGuard Fit column (2.1 mm × 150 mm, 1.7 µm). The flow rate was 350 µL/min with mobile phase A water/ACN (95%: 5% *v*/*v*) containing 10 mM ammonium acetate and mobile phase B water/ACN (5%: 95% *v*/*v*) containing 10 mM ammonium acetate. The mobile phase gradient program started at 5% A, held constant for 1 min, linearly increased to 25% A at 5 min, linearly increased to 40% A at 10 min, linearly increased to 55% A at 11.5 min, held constant until 14.5 min, and returned to starting conditions at 16 min with acquisition stopping at 20.5 min. The column and autosampler temperatures were kept at 40 °C and 5 °C, respectively. Ionization was performed using heated electrospray ionization (HESI) with a spray voltage of 4.5 kV for positive mode and 3.5 kV for negative mode. The vaporizer and the ion transfer tube temperatures were set at 350 °C and 325 °C, respectively. A sheath gas flow of 40 (arbitrary units) and a sweep gas flow of 2 (arbitrary units) were applied for acquiring data. SRMs (Selected Reaction Monitoring) were determined using authentic standards and metabolite identity was confirmed using a combination of 2 SRMs for most metabolites as well as retention times obtained from the analysis of the authentic standards. Peak integration was performed using Skyline [29] and exported as CSV in the form of a data matrix for further analysis.

To optimize data quality, we used the TSQ Altis Method Editor (version 3.4) to assign dwell time prioritization. Data from a pooled urine sample was first acquired using this method, and peak intensities for each metabolite were assigned in roughly equal proportions to five buckets, with 1 being the highest priority for the least abundant signals that require the highest dwell time and 5 the lowest priority for the most abundant signals requiring the least amount of dwell time. Based on this assignment, the software calculated the optimal distribution of dwell times while maintaining >10 data points per peak.

### 2.6. Chromatographic Conditions

The following columns were assessed: Atlantis Premier BEH Z-HILIC (BEH-HILIC, 2.1 mm × 150 mm × 1.7 µm VanGuardTM Fit), Acquity Premier BEH Amide (BEH-amide, 2.1 mm × 150 mm × 1.7 µm), and SeQuant ZIC-HILIC (zic-HILIC, 2.1 mm × 100 mm × 3.5 µm). The first two columns were each tested with mobile phases at pH 3, 7, and 10, whereas the zic-HILIC column was tested at pH 3 and 7. Mobile phase A composition for neutral pH consisted of water/ACN (95%: 5% *v*/*v*) containing 10 mM ammonium acetate, and for mobile phase B water/ACN (5%: 95% *v*/*v*) containing 10 mM ammonium acetate was used. For acidic and basic conditions, mobile phase A and B were modified with acetic acid and ammonium hydroxide, respectively.

For each of 145 metabolite standards, chromatographic quality was scored based on (1) retention (>3.8 min is good (green), 2–3.8 min is acceptable (yellow), and <2 min unacceptable (red)); (2) peak quality (narrow and symmetric is good (green), slight tailing or fronting is acceptable (yellow), and split or excessively broad peaks is unacceptable (red)); and (3) MS response (strong MS response is good (green), MS signal < half of the highest observed is acceptable (yellow), and very low signals are unacceptable (red)). Each metabolite was assigned one color based on the lowest scoring attribute for each column and mobile phase pH combination.

### 2.7. Data and Statistical Analysis

#### 2.7.1. Analysis of the Impact of U-^13^C Metabolite Yeast Extract on Linearity of Response and Normalization

Ratios of endogenous (^12^C) and U-^13^C metabolites were calculated based on the internal and surrogate standards in Supplementary Table S1. Correlations between metabolite raw peak intensities or ratios and relative concentration levels were determined for each metabolite using Pearson’s correlation coefficient.

#### 2.7.2. Assessment of Within- and Across-Batch Precision Using QC Samples

Data from aliquots of the same pooled urine sample from 3 separate datasets (3–4 samples per dataset) were collected over a six-month period. For within-batch precision, ratios of endogenous (^12^C) and U-^13^C metabolites were calculated. For across-batch precision, intensities of all (^12^C and U-^13^C) metabolites were first normalized to the mean of the sample’s batch to correct for batch differences prior to calculating ratios. The percent coefficient of variation (CV) for each metabolite ratio was calculated as follows:

$$CV(\%)=\frac{1}{\mu }\sqrt{\frac{\sum {\left(r_{i}-\mu \right)}^{2}}{\left(N-1\right)}}\times 100\%$$

in which $r_{i}$ is the vector metabolite ratio value of sample i; μ is the sample mean; and N is the sample size. The within-batch percent CV was computed for samples contained within individual datasets.

#### 2.7.3. Comparison with NMR Data to Evaluate Data Accuracy

A total of 225 urine samples from kidney transplant patients obtained either within 2 weeks or a year after transplant surgery were analyzed using 2D-NMR and LC-MS with Olaris. The NMR data were collected on a Bruker AVANCE II solution-state 600 MHz spectrometer equipped with a liquid helium-cooled Prodigy TCI Cryoprobe (H/F, C, N) using noesypr1d and hsqcetgpsisp2.2 pulse programs and non-uniform sampling (NUS). The acquired 2D spectral data were processed and reconstructed using iterative soft thresholding using the NMRPipe software package (version 11.2), and 2D-NMR metabolite features were identified using Olaris’ 2D-NMR analysis pipeline [30]. The correlation between raw 2D-NMR and LC-MS intensities was determined using Pearson’s correlation coefficient.

#### 2.7.4. OGP Method Performance in a Clinical Sample Study

The Skyline data output for the kidney transplant urine was processed by a dedicated pipeline. Signal loss correction was implemented using QC locally estimated scatterplot smoothing (QC-LOESS) on the pooled biological urine samples to mitigate signal loss over the course of the sequence. Three times the mean intensity in blank samples served as the detection lower bound (DLB) after signal loss correlation, with any signal lower than the DLB set to 0. Additionally, metabolites with more than 20% missing values were removed from the analysis.

After data processing, ratios between the endogenous metabolites and their internal or surrogate U-^13^C standards were calculated. A post-acquisition normalization procedure was implemented to remove biological variation. Biological variation was estimated by calculating the median of all metabolite ratios of a sample. All metabolite ratios in that sample were then normalized by dividing them by the following median:

$$\alpha_{i}=median\left(r_{i}\right)$$

$$\underline{r_{i}}=\frac{r_{i}}{a_{i}}$$

where $\alpha_{i}$ is the biological variation estimated from all the metabolite ratios ($r_{i})$; $\underline{r_{i}}$ are the normalized metabolite ratios. Finally, standard deviation (SD) scaling and log transformation standardized the distribution of metabolite data.

A Kruskal–Wallis (KW) non-parametric one-way analysis of variance (ANOVA) and fold change (FC) was utilized to identify differential metabolites between experimental groups. FC was calculated as the ratio between medians of two groups:

$$FC=\frac{{\tilde{r}}_{a}}{{\tilde{r}}_{b}}$$

where ${\tilde{r}}_{a}$ and ${\tilde{r}}_{b}$ are median metabolite ratios of 2 groups. *p*-values from the KW test were adjusted for false discovery rate (FDR) for multiple hypothesis testing correction. A differential metabolite was defined as any metabolite having an FDR-adjusted *p*-value < 0.05 and an FC exceeding the 1.5 cutoff (FC values greater than 1.5 or less than 0.67 were considered indicative of increased or decreased levels, respectively).

All data processing and statistical analyses were performed using R (version 4.2.3).

## 3. Results

### 3.1. Optimization of Liquid Chromatography and Mass Spectrometry Parameters to Facilitate High-Quality Data Generation for Hundreds of Metabolites

**Figure 1:**
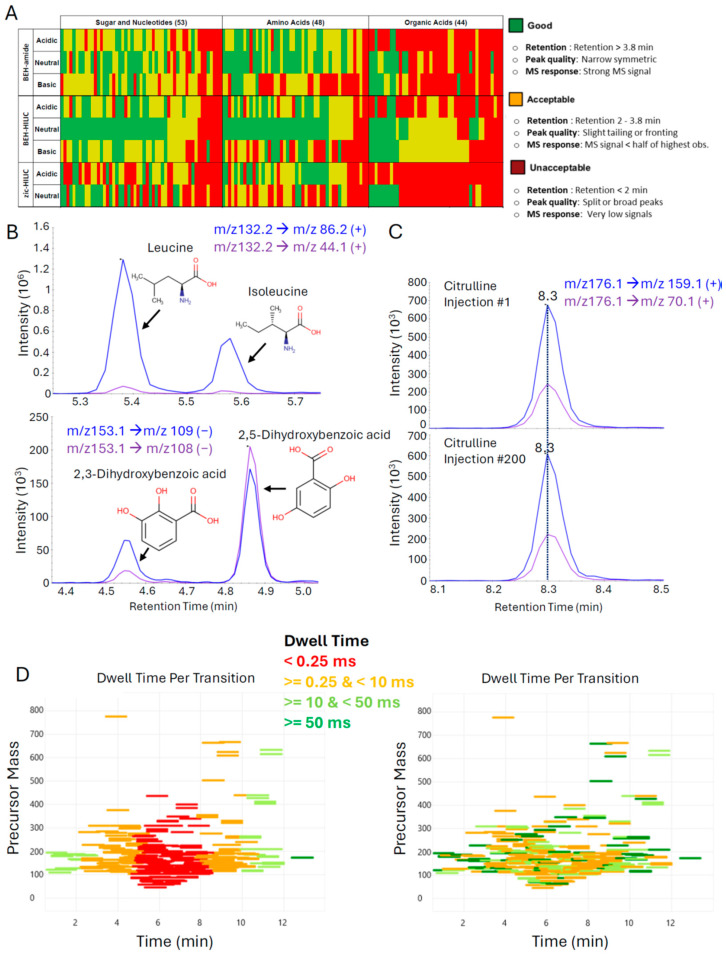
Optimizing chromatographic and MS conditions to facilitate at-scale metabolite measurements: (**A**) Comparison of separation performance for multiple columns and mobile phase conditions (pH). See main text and Section 2 for details. (**B**) Examples of baseline separation of isomers in a pooled urine sample. (**C**) Retention times remain stable for at least 200 LC-MS injections of pooled urine, as exemplified by citrulline. (**D**) Dwell time optimization to improve metabolomics data quality. MS instrument cycle time is affected by the peak width, number of desired datapoints for each peak, the set retention time window, and the number of scheduled SRMs per measurement cycle. More SRMs per measurement cycle means less dwell time per SRM, affecting data quality (left panel). Prioritizing dwell times based on metabolite signal abundance increases dwell time for low abundant metabolites to improve S/N, while minimizing time spent on high-intensity metabolites.

The purpose of the OGP is to generate accurate and reproducible data for polar metabolites that are routinely present in human plasma and urine. To generate a target list, we filtered the Human Metabolomics Database (www.hmdb.ca, accessed on 10 September 2022) for endogenous metabolites that have been detected and quantified in either or both matrices and removed the molecules classified as lipids [31]. This resulted in a list of approximately 1200 metabolites. To minimize sample consumption, sample acquisition, and processing time, the next objective was to find a universal LC-MS method for the analysis of as many of these metabolites as possible. Due to the polar nature of the metabolites, we exclusively focused on HILIC columns. In total, 145 metabolite standards were acquired with a chemical diversity representative of the list of 1200; 48 were amino acid(-like) molecules, 44 were organic acids, and the remaining 53 were nucleotides and sugars. Chromatographic performance of three HILIC columns and three mobile phase pH conditions (two for one column) was systematically assessed using mixtures of these standards. Specifically, the SeQuant ZIC-HILIC (zic-HILIC), the Atlantis Premier BEH Z-HILIC (BEH-HILIC), and the Acquity BEH Amide (BEH-amide) columns were tested using mobile phases at pH 3, 7, and 10 (the SeQuant column was only tested at pH 3 and 7, see Section 2 for details on columns and mobile phase composition). For each metabolite, chromatographic quality was scored based on retention, peak quality, and MS response. To present this data in a visually intuitive manner, each metabolite was assigned one color based on the lowest scoring attribute (retention, peak quality, MS response) for each column and mobile phase pH combination and the collective data integrated in a heatmap (Figure 1A). From this analysis, it was apparent the Atlantis Premier BEH Z-HILIC at pH 7 provided the best overall performance, in line with previous reports [32,33].

Following the selection of the column and mobile phases, additional standards were acquired, tuned, and analyzed by LC-MS to obtain SRM parameters and retention time, and the gradient optimized to maximize separation. At time of publication, the OGP contains SRMs for 236 endogenous metabolites covering all major metabolic pathways with more to be added in the future. Chromatographic quality was high with most peaks having <10 s peak widths and the ability to resolve isomer pairs, such as leucine-isoleucine and 2,3-dihydroxybenzoic acid-2,5-dihydroxybenzoic acid at baseline (Figure 1B). To evaluate the chromatographic stability of our final method, a pooled urine sample was injected 200 times and the retention times of the first and last sample were compared. Virtually no drift in retention times was observed, as illustrated by citrulline as a representative metabolite (Figure 1C).

Our method uses 2 SRMs for each metabolite to maximize confidence in annotation. As we also incorporate U-^13^C labeled metabolites as internal standards (see next Section 3.2), the total number of SRMs over the course of the approximately 13 min of separation exceeds 600. The reliable performance of the chromatographic approach permits the use of relatively tight, 1 min retention time windows in which the method scans for a particular metabolite. This drastically reduces the number of concurrent SRMs during each measurement cycle and, consequently, the time that can be spent on each transition increases (dwell time). Nonetheless, the number of concurrent SRMs remains high, peaking at around 6 min into the separation, with over 100 SRMs during a measurement cycle (Figure S1A). During this period, the dwell time per SRM dips well below 1 ms, impacting the quality of the data collected (Figure 1D, left panel). To optimize data quality, we used the ability of the instrument software to assign dwell time prioritization. Data from a pooled urine sample were first acquired by the method, and peak intensities for each metabolite were assigned in roughly equal proportions to five buckets, with one being the highest priority for the least abundant signals that require the highest dwell time and five the lowest priority for the most abundant signals requiring the least amount of dwell time. Based on this assignment, the software calculated the optimal distribution of dwell times while maintaining >10 data points per peak (Figure 1D, right panel). This significantly improves the data quality for low abundant signals, as illustrated by U-^13^C-guanosine (Figure S1B).

### 3.2. Impact of U-^13^C Metabolite Yeast Extract on Linearity of Response and Normalization

**Figure 2:**
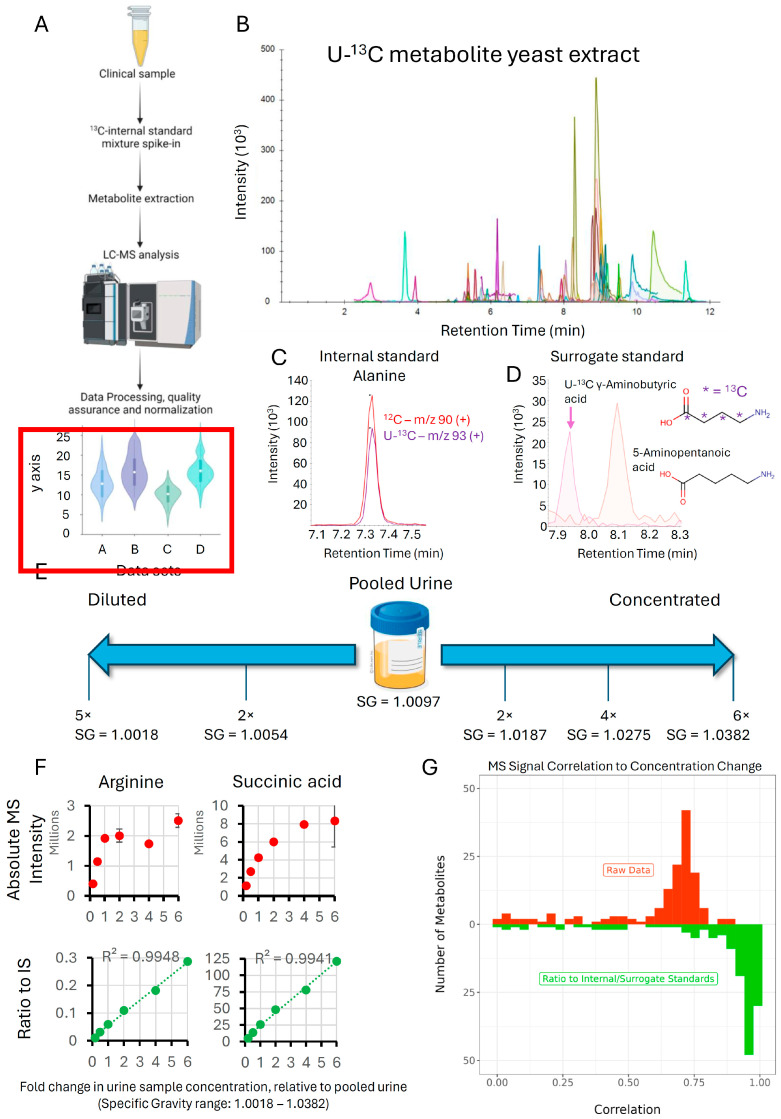
U-^13^C metabolite yeast extract improves linearity of response: (**A**) Spiking U-^13^C metabolite yeast extract into each sample prior to sample preparation and LC-MS analysis corrects for pre-analytical and analytical sources of variation and improves overall data quality. (**B**) LC-MS chromatogram of U-^13^C metabolite yeast extract. (**C**) Example chromatogram of an endogenous metabolite (alanine) from a pooled urine sample and the U-^13^C labeled standard from the spike yeast extract. (**D**) U-^13^C isotopologues of chemically similar metabolites can act as ‘surrogate’ standards for those metabolites lacking their own internal standard. Data is from a pooled urine sample. (**E**) Schematic illustrating how pooled urine was diluted and concentrated multiple times to mimic the range of specific gravity (SG, concentration of urinary solutes including metabolites) observed in the clinic. (**F**) Examples of how the ratio to internal standard (green) improves linearity of response compared to the absolute metabolite signal (red). Data points are averages of 3 replicate measurements and error bars are s.d. (**G**) Degree of correlation to a linear calibration curve of metabolite ratios to internal or surrogate standards vs absolute metabolite intensities (raw data) for all OGP metabolites.

A major consideration when establishing an accurate LC-MS method is the fact that the MS response is affected by a variety of factors in addition to analyte abundance. For instance, the response can vary significantly day-to-day depending on when and how the system was last cleaned and calibrated, what samples were run immediately prior to the current analysis, and the state of column. Additionally, even within a single batch, variations in pre-analytical (sample preparation) and analytical (ion suppression/enhancement due to matrix effects, decrease in sensitivity as the sequence progresses) factors impact data quality. Fortunately, these collective sources of error can be conveniently addressed by using internal standards. Stable isotope-labeled (^13^C, ^15^N, ^2^H) internal standards are particularly well-suited, as their chemical behavior is near-identical to their unlabeled, endogenous counterpart but can be distinguished by MS. If internal standards are spiked in immediately prior to sample preparation, they can be used to account for both the pre-analytical and analytical sources of error discussed above (Figure 2A).

While the use of labeled internal standards improves data quality, a key issue in metabolomics is the intractability of sourcing these standards for hundreds of metabolites, as they are expensive and making internal standard mixtures is cumbersome. A more feasible alternative is the use of commercially available U-^13^C metabolite yeast extract [34]. To generate this labeled metabolite extract, yeast cells are cultured in U-^13^C-glucose in highly controlled conditions and for a sufficiently long duration to ensure complete labeling of metabolites. Metabolite extracts from these yeast cells can then be spiked into each sample as a single source of internal standards.

To determine which yeast metabolites can be detected by the OGP, we first analyzed an unlabeled metabolite yeast extract. This resulted in the detection of 78 metabolites, with MS signal intensities ranging from close to the detection limit to very abundant. SRMs for the U-^13^C metabolites were then calculated using the structural information provided for the fragments by mzCloud (https://www.mzcloud.org/, accessed on 5 April 2023) and through manual structural elucidation of fragments for those metabolites that were not in the mzCloud database or lacked structural information. These U-^13^C SRMs were then added to the OGP and the presence of U-^13^C metabolites was confirmed by running the U-^13^C metabolite yeast extract (Figure 2B). These metabolites eluted in the 2–12 min range, which is also the time window where most endogenous metabolites elute and represented metabolites from all major metabolite classes (Figure S2A). As expected, spiking a pooled urine sample with the U-^13^C metabolite yeast extract facilitates the detection of both the endogenous (^12^C) and U-^13^C isotopologues of metabolites, as exemplified by alanine (Figure 2C). Of note, this and subsequent experiments revealed that, when the U-^13^C metabolite yeast extract is spiked into biological samples, the lower abundant U-^13^C metabolite signals tend to become lost due to ion suppression, with approximately 50 metabolites remaining with sufficient quality to be used as internal standards in most of our experiments. To expand the scope of use of these internal standards, we also considered using the U-^13^C isotopologues of chemically similar molecules as ‘surrogate standards’ for those metabolites without their own internal standard. For instance, there was no U-^13^C labeled isotopologue for 5-aminopentanoic acid in the yeast extract. However, there was one for γ-aminobutyric acid, which is chemically quite similar and has a comparable retention time (Figure 2D). Because of their structural similarity, these metabolites are likely to be affected similarly by matrix effects and we therefore reasoned that U-^13^C γ-aminobutyric would make for a suitable surrogate standard for 5-aminopentanoic acid. In a similar manner, surrogate standards were identified for all metabolites without a yeast extract internal standard based on structural similarity (Table S1).

The successful identification of biomarkers in a clinical sample stands or falls with the measurement accuracy of metabolite intensities, especially when fold changes between groups of interest are relatively subtle. Unfortunately, biomarker research is complicated by ion suppression/enhancement due to matrix effects. This is especially true for urine, where large sample-to-sample variations in sample concentration are the norm. To evaluate the impact of U-^13^C metabolite yeast spike-ins on data accuracy by improving linearity of response for clinical urine samples, we designed an experiment where pooled urine was diluted (2×, 5×) and concentrated (2×, 4×, 6×) multiple times (Figure 2E). The resulting concentration range simulates the range of specific gravities (SG, a measure of urine density) that is routinely observed for clinical urine samples, with SG = 1.0018 on the lowest end and SG = 1.0382 on the highest end of the spectrum. Urine samples at each concentration level were spiked with the same amount of U-^13^C metabolite extract as described in the Section 2 section. While a linear increase in metabolite signal intensity is expected as the metabolite concentrations increase from the least to the most concentrated sample, this was not observed for the absolute MS signal, especially at the higher end of the concentration range where ion suppression due to matrix effects is strongest, as illustrated by metabolites representing the major chemical classes (Figure 2F and Figure S2B, note that arginine showed particularly non-linear behavior). However, when MS signals were expressed as a ratio to their respective internal standards, linearity was maintained across the entire 30-fold concentration range with >0.994 correlation (r^2^) for the examples shown. An evaluation across all the metabolites in the OGP revealed a much higher correlation to a linear calibration curve for metabolite ratios to their internal or a surrogate standard compared to absolute MS signals (raw data, Figure 2G). As expected, when comparing metabolite ratios to internal standards and metabolite ratios to surrogates, ratios to internal standards demonstrated the highest correlation (Figure S2C). This demonstrates that the use of internal standards greatly improves measurement accuracy, improving the odds of identifying new biomarkers.

### 3.3. Assessment of Within- and Across-Batch Precision Using QC Samples

**Figure 3:**
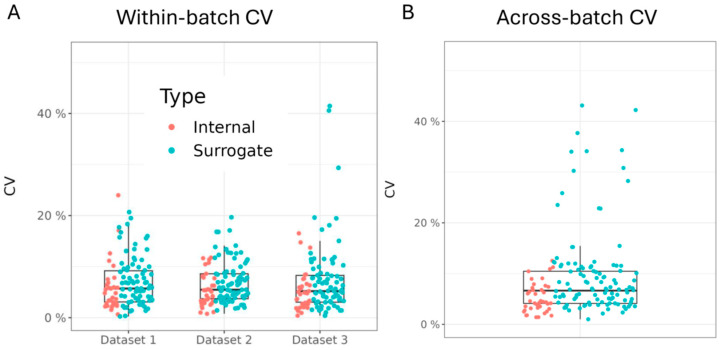
High within- and across-batch precision for metabolite ratios: (**A**) Within-batch CVs for all metabolite ratios to internal (red) or surrogate (teal) standards in a pooled urine sample for 3 individual datasets. CV is calculated based on 3–4 replicated measurements. (**B**) Across-batch CV for all metabolite ratios to internal or surrogate standards for the same pooled urine sample acquired in each of the 3 separate datasets acquired in a six-month period.

An important objective of the OGP is to provide highly precise and reproducible data, as this facilitates reliable comparison and mining of large datasets acquired over multiple batches. To evaluate both within- and across-batch coefficients of variation (CV), data from a pooled urine sample were collected in triplicate during the acquisition of each of three datasets collected over the span of six months. For all metabolites in the OGP, ratios to their internal standards or surrogate standards were calculated and directly compared. This demonstrated that the median within-batch CV for metabolite ratios to their internal standard was approximately 5% for all three datasets, and for metabolites without their own internal standard, the median CV of ratios to surrogates was 7–8% (Figure 3A). Next, the across-batch CV was calculated for the pooled sample acquired across the three datasets over a six-month period. The median CV was less than 10% for all metabolite ratios, with the median for the ratios to internal standards being 5% and the ratios to surrogates 11% (Figure 3B). Apart from Nightingale, which is an NMR-based platform with significantly lower metabolite coverage, the across-batch precision of the OGP outcompetes Biocrates (14%), HMT (12%), and Metabolon (18%) (Table S2) [2]. 

### 3.4. Comparison with NMR Data to Evaluate Data Accuracy

**Figure 4:**
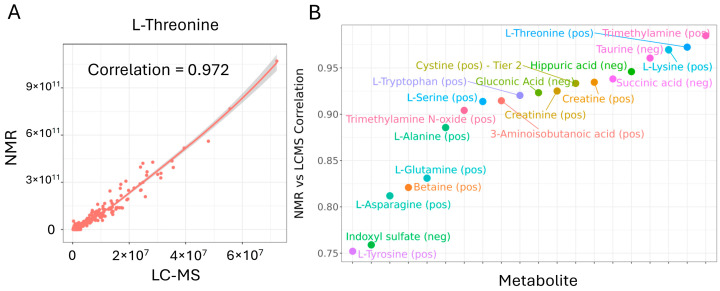
Comparison to 2D-NMR reveals high data accuracy for overlapping metabolites: (**A**) Correlation analysis of 2D-NMR and OGP LC-MS absolute signal abundances is performed across a set of >200 urine study samples. Neither the NMR nor the LC-MS data was adjusted or normalized in any way. (**B**) Correlation between NRM and LC-MS for metabolites that overlap between both platforms.

After confirming the linear response and precision of the OGP, we next sought to interrogate its accuracy in a biological sample set. As NMR is universally considered to be the gold standard in data accuracy, we exploited the fact that we use both NMR and LC-MS and therefore can directly compare the performance using samples measured on both platforms. Correlation analysis was performed on data collected from a large (>200) set of clinical urine samples with both 2D-NMR and the OGP [30]. This analysis was performed for metabolites that were measured on both platforms and for which the features on the NMR were unique and confirmed for the metabolite of interest. For each metabolite, the absolute signal intensities across all the samples as acquired by NMR and LC-MS were correlated, as illustrated by L-threonine (Figure 4A). As these measurements are a direct correlation of the distribution of signal intensities across the same set of samples measured by both platforms, and not a biological comparison, neither set of data was normalized in any way. Twenty metabolites met the above criteria and overlapped in the NMR and LC-MS datasets. For all of these, the correlation ranged from high (r^2^ = 0.75) to very high (r^2^ = 0.98) (Figure 4B). This strong agreement between the two platforms for these overlapping metabolites demonstrates the high accuracy of the OGP.

### 3.5. OGP Method Performance in a Clinical Sample Study

**Figure 5:**
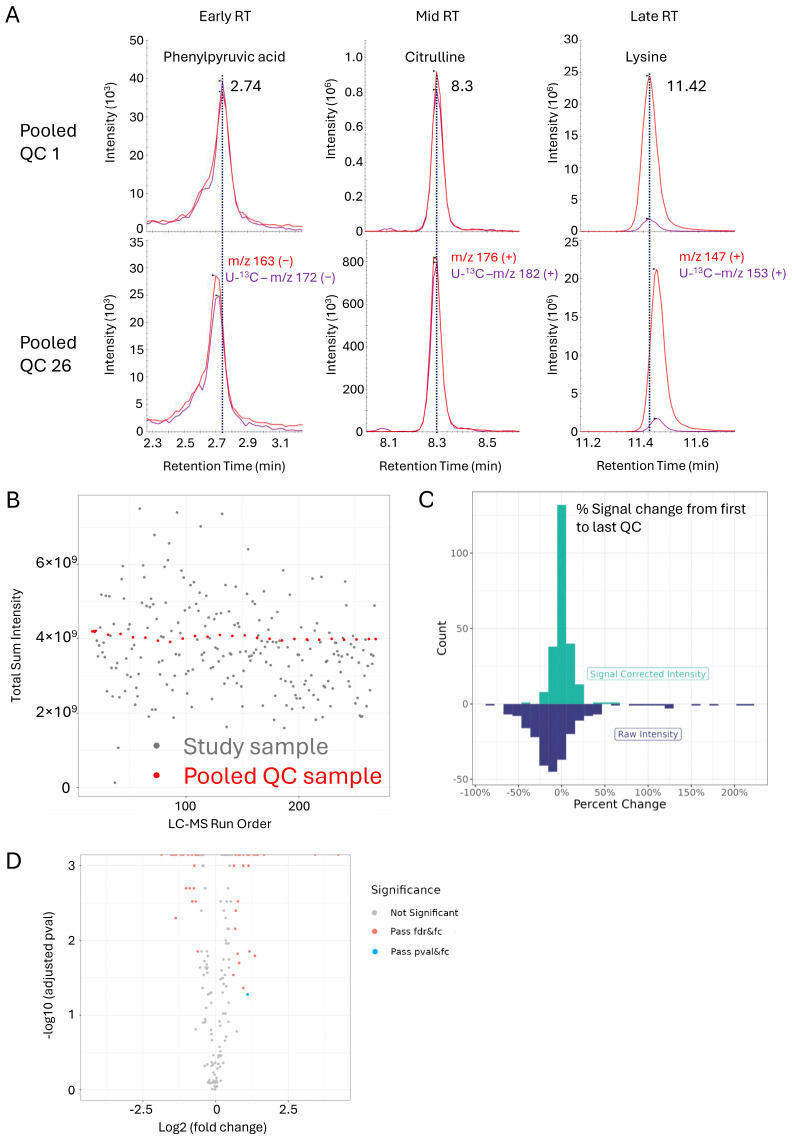
OGP performance for a batch of study samples: (**A**) Assessment of retention time fidelity across the batch comparing the first and last pooled QC samples, for representative early (phenylpyruvic acid), mid (citrulline), and late (lysine) eluting metabolites. (**B**) Robustness of sample MS signal intensities (shown is total sum intensities of all metabolites per sample) shown in the order the samples were run. The pooled QC injections are highlighted in red. (**C**) Histogram of distribution of metabolite MS signal changes comparing the first and the last pooled QC injection, for the raw data (blue) and signal corrected data (green, see Section 2 for details). (**D**) Volcano plot showing differences in metabolite intensity ratios for urine samples collected within two weeks versus one year post kidney transplant.

We next evaluated the performance of the OGP to detect clinically relevant metabolite signatures within a set of clinical samples. LC-MS analysis was performed on urine samples from patients who had received a kidney transplant with the objective of finding significant urine metabolite changes associated with time post-transplant. In total, 225 urine samples, obtained either within two weeks or a year after transplant surgery, were spiked with U-^13^C metabolite yeast extract and sample preparation was performed as described. LC-MS acquisition was performed in a single batch in randomized order interspersed with a pooled urine QC sample run after every 10 study samples. Comparison of the retention times of representative early, mid, and late eluting metabolites in the first versus the last QC sample run showed negligible drift of less than 6 s (Figure 5A), reaffirming the robustness of our HILIC-based method. Evaluation of summed metabolite MS signals for the samples in the order they were acquired showed significant variability from study sample to study sample (Figure 5B). This is caused by differences in sample concentration or specific gravity, which dictate overall metabolite abundance and points to the need to perform normalization prior to data analysis. Importantly, the repeated pooled QC sample runs showed consistent MS responses across the batch. A deeper, per metabolite, MS signal evaluation over the course of the batch revealed some signal loss for most metabolites (Figure 5C). This is a common occurrence and can be easily corrected as demonstrated by comparing the QC runs and this is why QCs should be included in each study. To determine if there was a metabolite signature differentiating transplant patients across time, the dataset was normalized. Importantly, median normalization was performed on the metabolite ratios to internal/surrogate standards rather than on absolute endogenous metabolite signals. This has the fundamental advantage that metabolite signals are adjusted for signal suppression/enhancement as the internal standards and endogenous molecules will be similarly affected, and we found this method to improve the accuracy of normalization (Figure S3). Post-normalization comparison of urine samples collected within two weeks or a year post-transplant surgery revealed multiple metabolites that reached our fold-change and statistics threshold (Figure 5D). Thus, our LC-MS method enables detection of clinically relevant metabolite signatures. 

## 4. Discussion

We live in an exciting era where it is possible to profile patient samples with unprecedented granularity using a variety of omics modalities (i.e., multi-omics). Moreover, the advent of new computational approaches, such as artificial intelligence (AI), provides new ways to detect patterns in the obtained data, segment patients, and generate biological hypotheses in ways that augment human knowledge. With some of the top diagnostics being metabolite-based, the clinical importance of metabolite measurements is beyond contention. As such, metabolomics is poised to make a significant contribution to the developments in multi-omics and precision medicine. At the same time however, technical challenges in metabolomics limit widespread adoption by the research community. Chief among these are (1) a lack of reproducibility in metabolite coverage and abundances across large sample sets, complicating interpretation and integration with other omics sets [2], and (2) a need for increased speed and ease of data acquisition and processing to match the scale at which other omics modalities can be performed. The lack of reproducibility is especially problematic as the output from computational modeling and AI will only be as good as the quality of the data provided.

Numerous efforts have been instigated to address some of the issues underlying the poor reproducibility, including standardized annotation [31], reporting [35,36], and tools for depositing data [37,38]. The OGP described in this paper directly addresses the limited reproducibility by using U-^13^C internal standards. When spiked into samples as an immediate first step, internal standards can be used to correct for the cumulative error introduced by sample preparation and LC-MS acquisition, including ion suppression/enhancement occurring due to matrix effects, which can vary significantly between clinical samples. A key consideration for the use of internal standards for metabolomics applications is how to deal with the sheer number of metabolites. Building mixtures of stable isotope labeled internal standards is expensive and labor intensive. Instead, commercially available U-^13^C metabolite yeast extract provides a convenient alternative. We identified around 50 U-^13^C labeled metabolites that can be used as internal standards for metabolites in the OGP, covering a variety of chemical classes (Figure S2A) and enabling an impressive median precision of 5% across datasets acquired in a six-month time span for ratios to internal standards and 10% when including ratios to surrogate standards. As our panel continues to evolve and grow, we will add more SRMs from U-^13^C metabolites from the yeast and will explore the addition of stable isotope labeled standards to further improve the method’s reproducibility. The internal standards can also be used as ‘surrogate’ standards for the metabolites in our panel, for which a ^13^C-isotopologue is not present in the yeast metabolite extract, significantly improving linearity of response (Figure 2F,G) and accuracy of normalization (Figure S3). We consider the OGP to be ‘standardized’ in that each metabolite is expressed as a ratio to either its own internal standard or a surrogate standard. While it is possible to estimate metabolite concentrations based on our data, we find ratios to be sufficient for accurate comparison across batches over the course of many months/years. Fully quantitative metabolomics methods have also been published [39] and are available as kits and services by organizations like Biocrates and TMIC. The obvious advantage of quantitative methods is that sample metabolite concentrations can be compared to any other sample irrespective of the exact quantitation approach used. Major considerations, however, are that these methods are more involved as they typically require derivatization and more experimental steps to determine concentrations, and that including new metabolites is usually non-trivial. The OGP on the other hand, is fast as it requires minimal sample prep, and flexible as new metabolites can be readily added. The choice in methodology should be guided by the research objectives.

Combining QqQ MS with U-^13^C internal standards provides data with exquisite accuracy and range of linear dynamic response. It also addresses the second key issue mentioned above, i.e., the need for increased speed and ease of use; the data that are being generated can be easily integrated and annotated, as each metabolite has its own assigned SRMs and a known retention time. This makes the data processing straightforward compared to untargeted metabolomics efforts using high-resolution data and is amenable to automation and implementation across labs with varying degrees of metabolomics expertise. Naturally, a disadvantage of using QqQ MS is that only the panel of metabolites for which SRMs have been included are being measured. As such, unknown metabolites cannot be detected. As with any experiment involving measurements, the choice of method depends on the context of use: in purely discovery/exploratory metabolomics studies, untargeted methods can be more appropriate. In scenarios where the chances of identifying unknowns are relatively low, and where ultra-high-quality measurements across large sample sets is essential, as is the case for clinical samples, QqQ MS is the technology of choice.

To ensure annotation fidelity, it is important to be mindful of interferences from other metabolites, i.e., when a signal in a metabolite’s SRM is caused by another metabolite. These are typically caused by in-source fragments from other metabolites, isomers, and isobars. Of note, except for isobars, these interferences also occur in high-resolution MS instruments. Luckily, many interfering metabolite combinations are known [40]. Cross-comparison of these published interferences with our own method, however, made us realize that interfering metabolites, by their very nature, must co-elute and therefore are highly dependent on the chromatographic conditions. We found that a good way to address this is to perform a correlation analysis of the abundances of all metabolite combinations across a set of study samples; for metabolite pairs with (near) perfect correlation the signal of one may actually derive from the other metabolite, requiring further scrutiny.

Our separation is based on HILIC chromatography. Following a comparison of multiple HILIC types and columns, and mobile phases with low, mid, and high pH, we identified ZIC-HILIC in combination with neutral pH mobile phases to provide the best overall performance across all major metabolite classes. This is in line with previously reported findings [32,33]. HILIC chromatography has a reputation for suffering from lower resolution relative to reverse-phase (RP) chromatography and for being less robust and reproducible. However, it has improved considerably over the years to the point where peak widths are now rivaling those obtained with RP separations and where both peak shape deterioration and retention time shift are virtually non-existent during a run and minimal between columns. Our method does include a considerable column wash and re-equilibration step (approximately a third of the run time per sample). However, this is easily remedied by introducing a column switching setup, where samples are injected interchangeably onto one of two columns while the other is regenerating from the previous run.

In summary, the OGP is a highly accurate and reproducible LC-MS metabolomics method. In this paper, the focus is on urine, as a primary interest in our laboratory is in monitoring kidney function. It is also for this reason that the OGP comprises several metabolite biomarkers of kidney function. These include amino acids and derivatives, such as asymmetric and symmetric dimethylarginine, phenylalanine, tyrosine, and known uremic toxins, such as indoxyl sulfate, all of which are differently metabolized and/or cleared when kidney function is compromised [41,42,43,44]. However, we have also successfully applied it to other matrices, such as plasma. The approach described here directly addresses three key limitations in clinical metabolomics that prevent a more widespread adoption alongside other omics in precision medicine applications, namely a lack of reproducibility in MS-based methods, ease of use, and portability to non-expert laboratories. The ability to rapidly generate and process highly accurate and reproducible metabolomics data will lower the burden for more widespread use in clinical applications and precision medicine.

## 5. Conclusions

Mass spectrometry (MS)-based metabolomics has the potential to impact clinical decision making. However, current methods are hampered by limited accuracy and precision. We developed the Olaris Global Panel (OGP), a HILIC LC-QqQ MS method leveraging U-^13^C metabolite yeast extract, for the comprehensive analysis of ~250 metabolites from all major metabolic pathways in clinical samples. This method achieved high (r^2^ > 0.90) goodness of fit to linear calibration curves for most metabolites across the clinical concentration range of urine samples and showed high correlation with NMR data from the same samples. Median within-batch CVs for all metabolite to internal standard ratios were consistently lower than 7% and less than 10% across batches that were acquired six months apart.

## Data Availability

Data is contained within the article or Supplementary Material.

## Supplementary Materials

The following supporting information can be downloaded at: https://www.mdpi.com/article/10.3390/metabo14050280/s1. Figure S1: Optimizing chromatographic and MS conditions to facilitate at scale metabolite measurements. Figure S2: U-^13^C metabolite yeast extract improves linearity of response. Figure S3: OGP performance for a batch of study samples. Table S1: List of panel metabolites and their internal/surrogate standards. Table S2: Overview of metabolomics vendors, their technology and across-batch CV.
