# Supplementary material for: Olaris Global Panel (OGP): A Highly Accurate and Reproducible Triple Quadrupole Mass Spectrometry-Based Metabolomics Method for Clinical Biomarker Discovery
Source: Metabolites. 2024 May 11;14(5):280. doi: 10.3390/metabo14050280 (PMC11123370; doi:10.3390/metabo14050280)
Supplement: Supplementary file 1 [file metabolites-14-00280-s001.zip › Dorrani_etal_SupplementalFiguresTable_51024.pptx]

## Slide 1
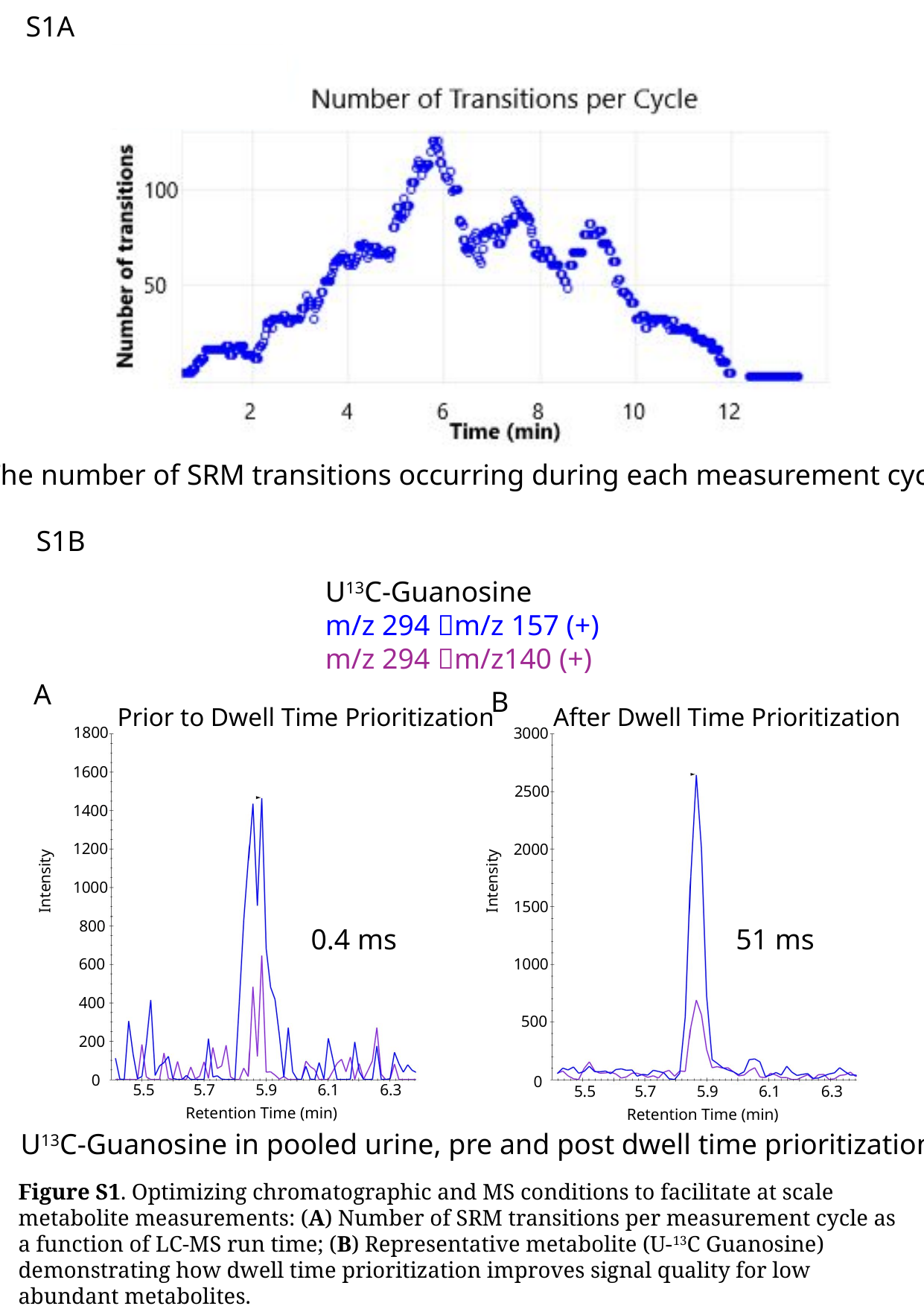

S1A
The number of SRM transitions occurring during each measurement cycle
S1B
U13C-Guanosine
m/z 294 m/z 157 (+)
m/z 294 m/z140 (+)
A
B
Prior to Dwell Time Prioritization
After Dwell Time Prioritization
1800
3000
1600
2500
1400
1200
2000
Intensity
Intensity
1000
1500
800
0.4 ms
51 ms
600
1000
400
500
200
0
0
5.5
5.7
5.9
6.1
6.3
5.5
5.7
5.9
6.1
6.3
Retention Time (min)
Retention Time (min)
U13C-Guanosine in pooled urine, pre and post dwell time prioritization
Figure S1. Optimizing chromatographic and MS conditions to facilitate at scale metabolite measurements: (A) Number of SRM transitions per measurement cycle as a function of LC-MS run time; (B) Representative metabolite (U-13C Guanosine) demonstrating how dwell time prioritization improves signal quality for low abundant metabolites.

## Slide 2
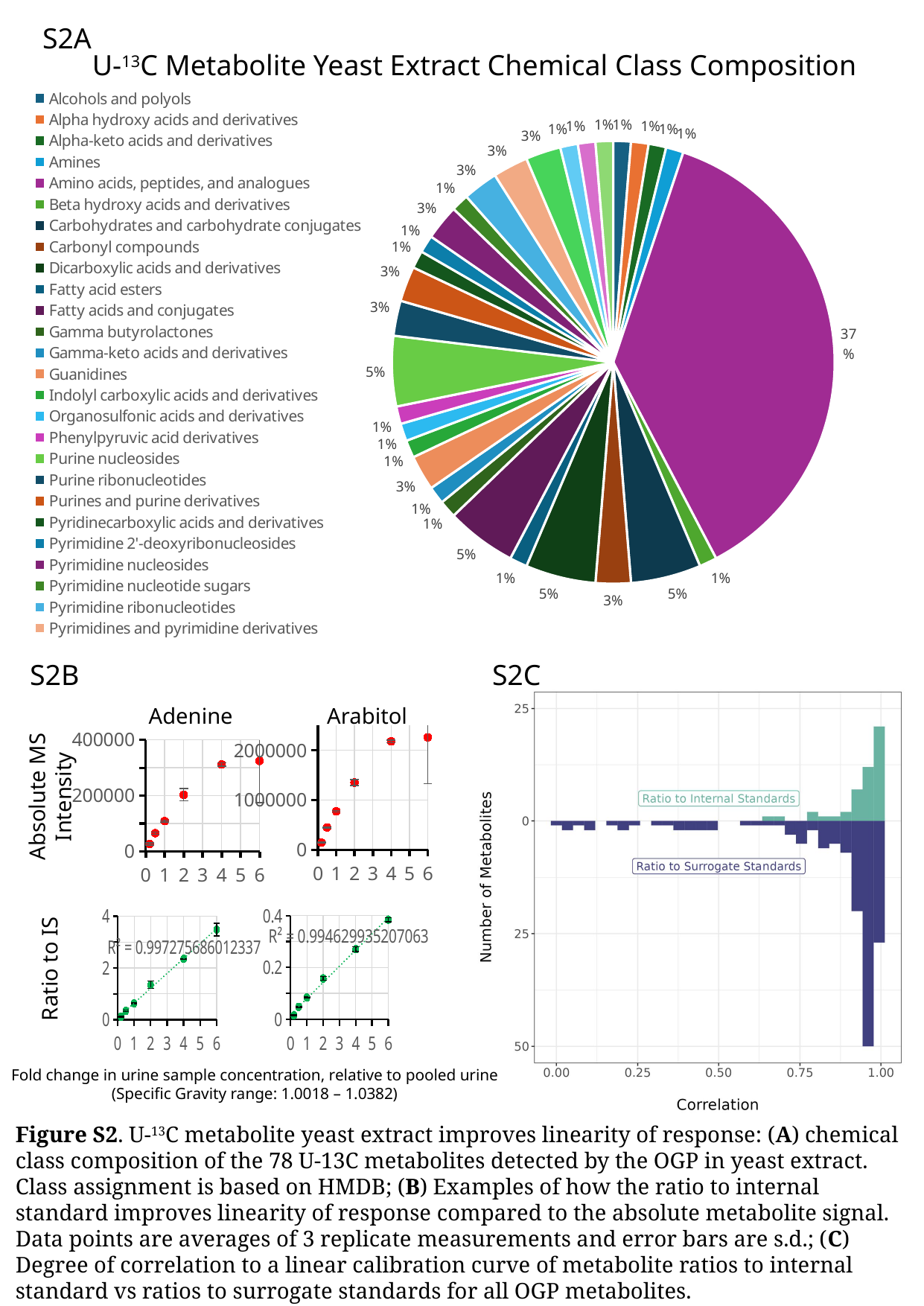

S2A
U-13C Metabolite Yeast Extract Chemical Class Composition
### Chart
| Category | |
|---|---|
| Alcohols and polyols | 1.0 |
| Alpha hydroxy acids and derivatives | 1.0 |
| Alpha-keto acids and derivatives | 1.0 |
| Amines | 1.0 |
| Amino acids, peptides, and analogues | 29.0 |
| Beta hydroxy acids and derivatives | 1.0 |
| Carbohydrates and carbohydrate conjugates | 4.0 |
| Carbonyl compounds | 2.0 |
| Dicarboxylic acids and derivatives | 4.0 |
| Fatty acid esters | 1.0 |
| Fatty acids and conjugates | 4.0 |
| Gamma butyrolactones | 1.0 |
| Gamma-keto acids and derivatives | 1.0 |
| Guanidines | 2.0 |
| Indolyl carboxylic acids and derivatives | 1.0 |
| Organosulfonic acids and derivatives | 1.0 |
| Phenylpyruvic acid derivatives | 1.0 |
| Purine nucleosides | 4.0 |
| Purine ribonucleotides | 2.0 |
| Purines and purine derivatives | 2.0 |
| Pyridinecarboxylic acids and derivatives | 1.0 |
| Pyrimidine 2'-deoxyribonucleosides | 1.0 |
| Pyrimidine nucleosides | 2.0 |
| Pyrimidine nucleotide sugars | 1.0 |
| Pyrimidine ribonucleotides | 2.0 |
| Pyrimidines and pyrimidine derivatives | 2.0 |
| Quaternary ammonium salts | 2.0 |
| Quinoline carboxylic acids | 1.0 |
| Short-chain hydroxy acids and derivatives | 1.0 |
| Short-chain keto acids and derivatives | 1.0 |S2B
S2C
Adenine
Arabitol
### Chart
| Category | |
|---|---|
### Chart
| Category | |
|---|---|Absolute MS
Intensity
### Chart
| Category | AVG Ratio |
|---|---|
### Chart
| Category | AVG Ratio |
|---|---|Ratio to IS
Fold change in urine sample concentration, relative to pooled urine
(Specific Gravity range: 1.0018 – 1.0382)
Figure S2. U-13C metabolite yeast extract improves linearity of response: (A) chemical class composition of the 78 U-13C metabolites detected by the OGP in yeast extract. Class assignment is based on HMDB; (B) Examples of how the ratio to internal standard improves linearity of response compared to the absolute metabolite signal. Data points are averages of 3 replicate measurements and error bars are s.d.; (C) Degree of correlation to a linear calibration curve of metabolite ratios to internal standard vs ratios to surrogate standards for all OGP metabolites.

## Slide 3
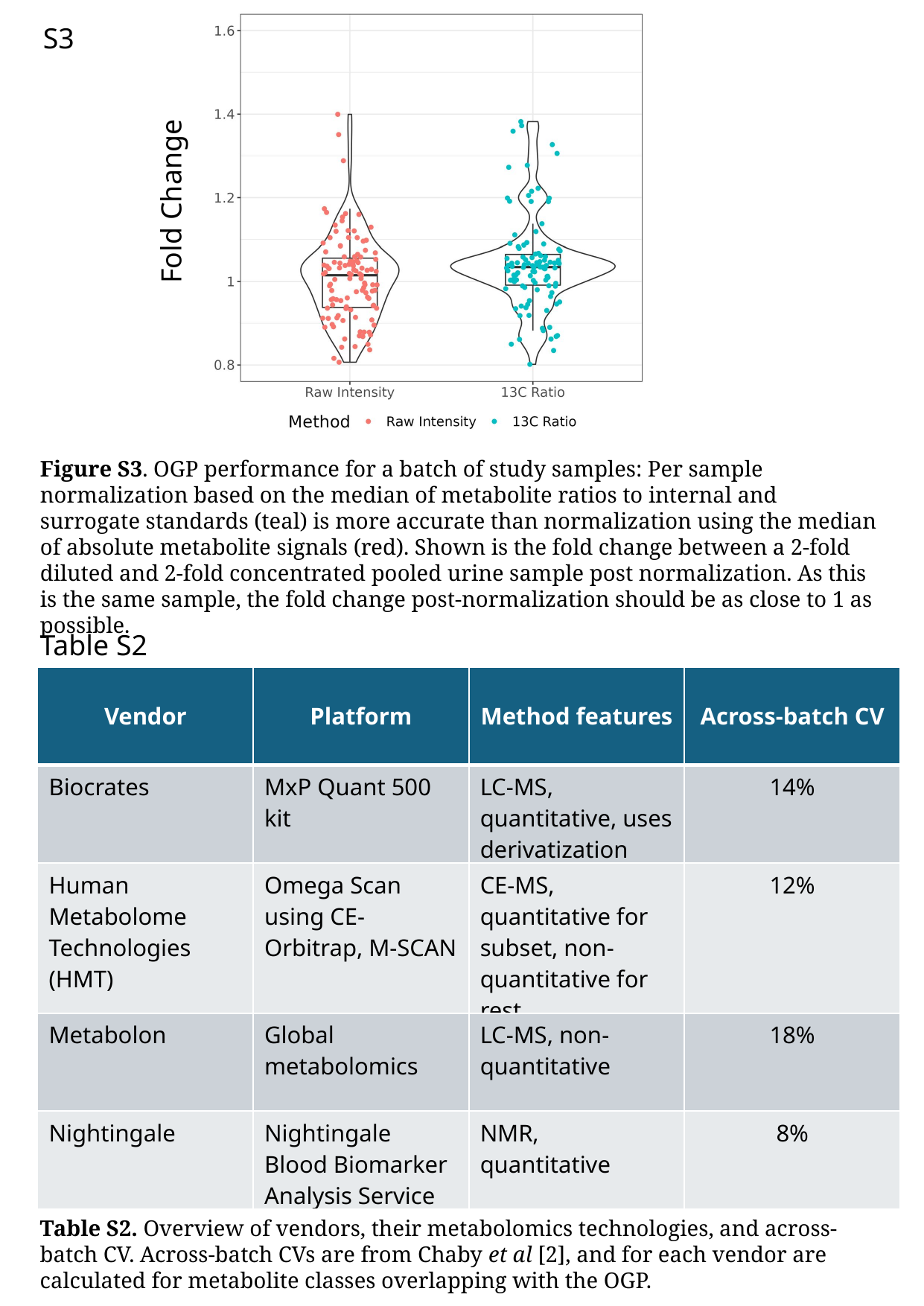

S3
Fold Change
Figure S3. OGP performance for a batch of study samples: Per sample normalization based on the median of metabolite ratios to internal and surrogate standards (teal) is more accurate than normalization using the median of absolute metabolite signals (red). Shown is the fold change between a 2-fold diluted and 2-fold concentrated pooled urine sample post normalization. As this is the same sample, the fold change post-normalization should be as close to 1 as possible.
Table S2
| Vendor | Platform | Method features | Across-batch CV |
| --- | --- | --- | --- |
| Biocrates | MxP Quant 500 kit | LC-MS, quantitative, uses derivatization | 14% |
| Human Metabolome Technologies (HMT) | Omega Scan using CE-Orbitrap, M-SCAN | CE-MS, quantitative for subset, non-quantitative for rest. | 12% |
| Metabolon | Global metabolomics | LC-MS, non-quantitative | 18% |
| Nightingale | Nightingale Blood Biomarker Analysis Service | NMR, quantitative | 8% |
Table S2. Overview of vendors, their metabolomics technologies, and across-batch CV. Across-batch CVs are from Chaby et al [2], and for each vendor are calculated for metabolite classes overlapping with the OGP.
